# Unmanned aerial systems-based remote sensing for monitoring sorghum growth and development

**DOI:** 10.1371/journal.pone.0196605

**Published:** 2018-05-01

**Authors:** Sanaz Shafian, Nithya Rajan, Ronnie Schnell, Muthukumar Bagavathiannan, John Valasek, Yeyin Shi, Jeff Olsenholler

**Affiliations:** 1 Department of Soil and Crop Sciences, Texas A&M University, College Station, Texas, United States of America; 2 Department of Aerospace Engineering, Texas A&M University, College Station, Texas, United States of America; 3 Department of Biological and Agricultural Engineering, Texas A&M University, College Station, Texas, United States of America; 4 Department of Geography, Texas A&M University, College Station, Texas, United States of America; Instituto Agricultura Sostenible, SPAIN

## Abstract

Unmanned Aerial Vehicles and Systems (UAV or UAS) have become increasingly popular in recent years for agricultural research applications. UAS are capable of acquiring images with high spatial and temporal resolutions that are ideal for applications in agriculture. The objective of this study was to evaluate the performance of a UAS-based remote sensing system for quantification of crop growth parameters of sorghum (Sorghum bicolor L.) including leaf area index (LAI), fractional vegetation cover (f_c_) and yield. The study was conducted at the Texas A&M Research Farm near College Station, Texas, United States. A fixed-wing UAS equipped with a multispectral sensor was used to collect image data during the 2016 growing season (April–October). Flight missions were successfully carried out at 50 days after planting (DAP; 25 May), 66 DAP (10 June) and 74 DAP (18 June). These flight missions provided image data covering the middle growth period of sorghum with a spatial resolution of approximately 6.5 cm. Field measurements of LAI and f_c_ were also collected. Four vegetation indices were calculated using the UAS images. Among those indices, the normalized difference vegetation index (NDVI) showed the highest correlation with LAI, f_c_ and yield with R^2^ values of 0.91, 0.89 and 0.58 respectively. Empirical relationships between NDVI and LAI and between NDVI and f_c_ were validated and proved to be accurate for estimating LAI and f_c_ from UAS-derived NDVI values. NDVI determined from UAS imagery acquired during the flowering stage (74 DAP) was found to be the most highly correlated with final grain yield. The observed high correlations between UAS-derived NDVI and the crop growth parameters (f_c_, LAI and grain yield) suggests the applicability of UAS for within-season data collection of agricultural crops such as sorghum.

## Introduction

In recent years, unmanned aerial vehicles (UAV) or systems (UAS) are gaining significant popularity as a potential technology that can acquire remote sensing imagery with ultra-high spatial resolution by flying at low altitude [1]. Since early 2015, the Federal Aviation Administration (FAA) has granted over 5500 petitions for commercial operation of UAS in the United States [2]. The Association of Unmanned Vehicle Systems International (AUVSI) predicts that 80% of these UAS will be used in agriculture. Compared with other remote sensing platforms such as satellites and manned aircrafts, UAS can be deployed easily and have lower operational cost, making them a promising tool for frequent monitoring of agricultural research sites and farmers’ fields. In the United States, the UAS technology is projected to generate more than $82 billion for the economy in the coming years [1].

Commonly used UAS in agricultural research are fixed-wing or rotorcraft systems. Both classes of vehicle have unique characteristics that make them useful for specific field-based agricultural applications [3]. Rotorcraft UAS can vertically take off and land (VTOL) so that the space requirements for take-off and landing are small. These types of UAS can also hover above an item of interest which makes them enable to take clear images of selected areas. This capability coupled with a very low forward speed when needed for imaging purposes are ideal for collecting ultra-high resolution images, highly detailed plant measurements, and plant health monitoring [4]. However, because the electric motor must directly lift the battery packs in addition to the sensor, these systems often have low flight speed and short flight ranges which limits their coverage areas. In contrast, fixed-wing UAS are equipped with wings for lifting that make the system suitable for mapping larger areas by covering long distances [5]. One disadvantage of the fixed-wing UAS is the need for a runway or launcher for takeoff and landing. Because fixed-wing UAS require air moving over their wings to generate lift, they must stay in a constant forward motion and cannot stay stationary the same way a rotary-wing UAS can. Regardless of the class of UAS used, a range of customizable sensors can be integrated for agricultural studies. Multiple types of cameras and sensors such as regular off-the shelf digital cameras [6], custom-built multi-spectral cameras [7, 8], hyperspectral imaging systems [9], and thermal cameras [10] are gaining popularity for measuring spectral information using UAS.

In this study, we investigated the use of a fixed-wing UAS platform equipped with a Sentek GEMS 35 multispectral sensor for assessing the growth and development of sorghum (*Sorghum bicolor* L.), a major cereal crop. Leaf area index (LAI), plant height and fractional vegetation cover (f_c_) are some of the variables routinely used for monitoring crop growth and development [11–13]. Most of the commonly used methods for measuring these variables are field-based techniques conducted manually. Different remote sensing–based approaches are available to quantify plant physiological variables. However, the majority of these studies are based on satellite or aircraft remote sensing systems which produce coarse resolution images that are not suitable for small plot research studies [14–17]. The recent advancements in sensor technologies and availability of low cost UAS systems have promoted their use in row crop agricultural research. Hunt et al. [18] used an image acquisition system mounted on an unmanned helicopter to estimate biomass and nitrogen status for corn (*Zea mays* L), alfalfa (*Medicago sativa* L) and soybeans (*Glycine max* L). Swain et al [19] used a radio-controlled unmanned helicopter to acquire images to estimate grain yield and total biomass of rice (*Oryza sativa*). They demonstrated that rice grain yield and biomass are highly correlated with the Normalized Difference Vegetation Index (NDVI) estimated from images. Vega et al [20] showed that there is a good correlation between NDVI extracted from images acquired from a quadcopter and nitrogen content of sunflower (*Helianthus annus*). Zarco-Tejada et al [21] demonstrated the feasibility of a micro-hyperspectral imager and a light-weight thermal camera mounted on a small UAS platform to track stress levels in citrus orchards. Recently, Elarab et al [22] combined thermal and multispectral images obtained using a UAS called AggieAirTM to estimate chlorophyll content of oats (*Avena sativa*).

The overall goal of the present study was to investigate the application of remote sensing imagery acquired using a fixed-wing UAS for monitoring sorghum growth and development. Specifically we examined the relationship between UAS-based vegetation indices with sorghum growth parameters such as LAI and f_c_. We also examined the relationship between sorghum grain yield and UAS-based NDVI at specific dates.

## Methods and materials

### Study area

The study site is located at the Texas A&M AgriLife Research Farm near College Station, TX (30°32′ 29.54″ N, 96° 25′ 37.24″ W; 103 m elevation). The region has a humid subtropical climate with average annual precipitation of approximately 1000 mm [23]. Mean air temperature during the growing season in 2016 (April–August) was 20° C with total rainfall of 690 mm. The soil type at this location is ships clay (very-fine, mixed, active, thermic Chromic Hapluderts) with 0–1% slope. The experimental design was a randomized split plot design with three replications. The main plot treatments included three seeding rates (30,000, 60,000 and 90,000 seeds/acre) and subplot treatments included six grain sorghum hybrids (BH 4100, RV 9782, AG 1203, DKS 37–07, DKS 53–53 and M75GR47). In total, there were 54 plots (3 m wide and 15 m long). Grain sorghum seeds were planted on 5 April 2016 with a four-row John Deere 1705 vacuum planter equipped with precision seed meters calibrated to deliver the desired seeding rates. Row spacing was 0.76 m. All seeds were treated with Concep III (Fluxofenin, 0.4 g a.i. per kg seed) before planting. All treatments received 161 kg ha^-1^ ammonium polyphosphate (11-37-0) at planting and 100 kg ha^-1^ of urea ammonium nitrate (28-0-0; check) 60 days after planting (DAP). Plots were mechanically harvested with a John Deere 3300 plot combine integrated with the HarvestMaster™ Grain Gauge HM800™ after reaching physiological maturity on 31 August 2016.

### Unmanned aerial system

**Fig 1:**
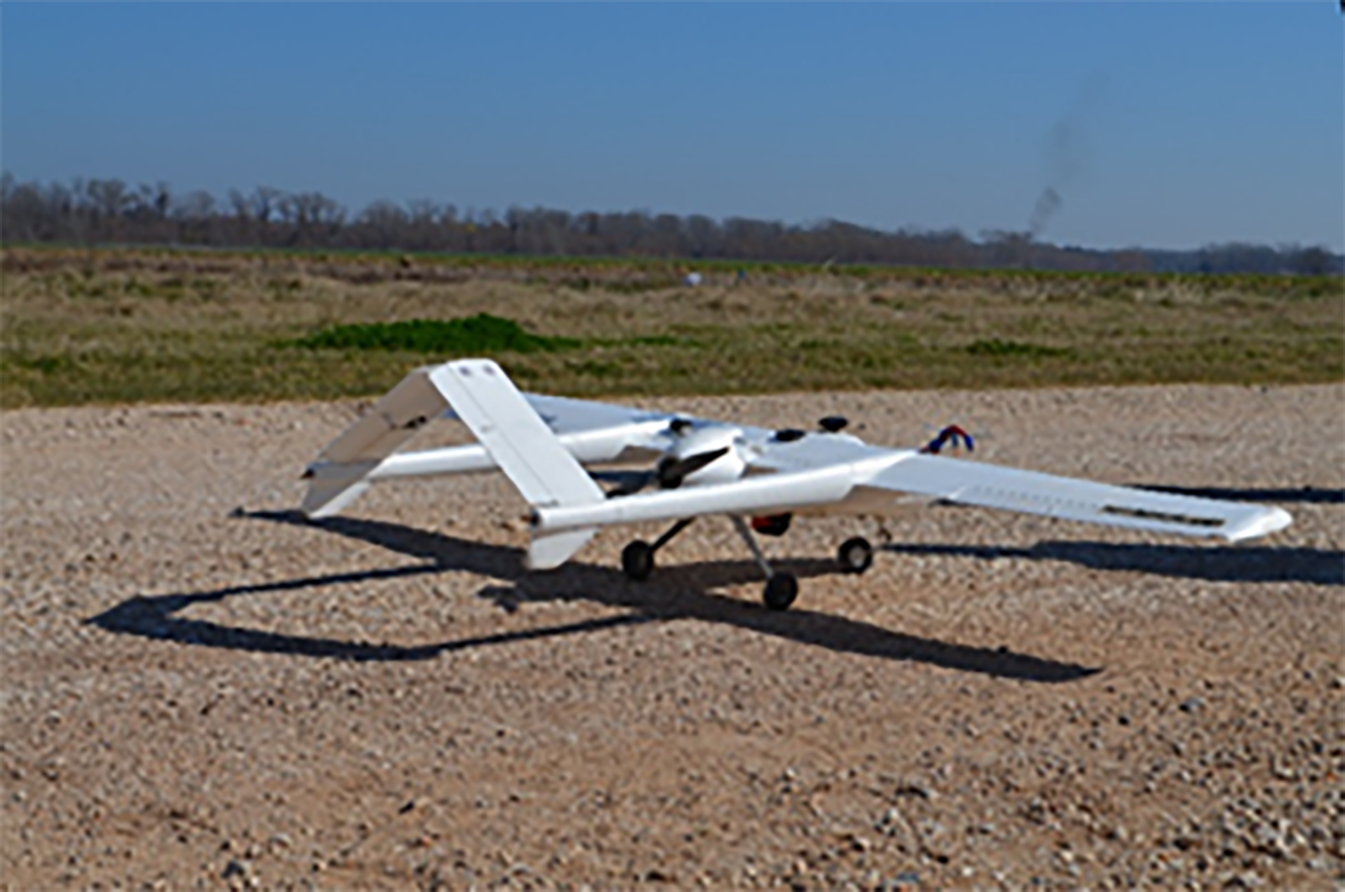
The unmanned aerial system ReadyMadeRC Anaconda.

In our study, we used a fixed-wing UAS (Model Anaconda, ReadyMadeRC, Lewis Center, Ohio, USA; www.readymaderc.com) as shown in Fig 1. This UAS was selected primarily for its low-cost (approximately $400), durable foam construction, and relatively large payload bay that can be easily retrofitted to accept almost any sensor. The Anaconda has an empty weight of 2.4 kg, a wingspan of 2.1 m, and can carry a payload of 1.8 kg for 45–60 minutes. Two 14.8v 5000 mAh lithium polymer battery (LiPo) packs were mounted in the nose in parallel to achieve 10,000 mAh of power. Major airframe modifications were also needed to mount the sensors in the correct positions. A center section of foam was removed from the underside of the fuselage, and a 3D-printed multi-hole mounting structure was installed. The mounting system is a light-weight and sturdy system that enables the capability of mounting multiple sensors and switching the sensors easily and quickly in the field while maintaining structural integrity. It ensures that at least two sensors can have a clear field of view of the ground at all times. Details of the mission design and the flights used to collect the data presented in this paper are contained in Valasek et al [5]. The sensor mounted on the UAS to acquire the imagery was a Sentek GEMS 35 multispectral camera (www.precisionaguavs.com) with an integrated GPS system. The camera acquires 1.2-megapixel images in four spectral bands (Near infrared [NIR], Red, Green, and Blue bands) with 8-bit radiometric resolution.

### Image acquisition and processing

Images were acquired within ± 2.0 hours of solar noon with flight duration ranging from 20 to 25 minutes under clear sky conditions. Flight (flight speed and path) and sensor parameters (exposure time, aperture, sensitivity, and frame rate) were selected to ensure that there was adequate overlap between images for mosaicking [3]. The sensor setting resulted in 75% forward overlap and 65% side overlap which was enough to generate good-quality mosaics. Flight altitude was 120 m above ground level. The flight planning used the “moving box” technique along with auto-triggering of the sensor [5]. Three flight missions were successfully performed in the growing season at 50 days after planting (DAP; 25 May), 66 DAP (10 June) and 74 DAP (18 June), providing image data covering the middle growth period of sorghum with spatial resolution of approximately 6.5 cm.

After multispectral image acquisition, pre-processing operations included mosaicking and radiometric calibration. Image mosaicking was performed using the Pix4Dmapper image analysis software (Pix4D SA, Lausanne, Switzerland), which combined all individual images together into a large georectified mosaic image for the entire study area. Radiometric calibration and all post-processing operations were performed using the image analysis software ENVI (Harris Geospatial, Boulder, CO, United States). Two 3 x 3 m reflectance reference tarps (Group Eight Technologies, Provo, UT, United States) with a nominal reflectance of 0.03 and 0.22 were used for radiometric calibration. Calibration tarps were laid adjacent to the study area during each flight mission. For each acquisition date, digital number (DN) values corresponding to the calibration tarps were extracted from the UAS imagery using ENVI. Linear regression equations were developed using tarp DN values and known reflectance values of the tarp in each spectral band [Eq 1]:

$$\rho_{\left(x,y,i\right)}=a_{i}\times {DN}_{\left(x,y,i\right)}+b_{i}$$ (1)

where *ρ*_*(x*,*y*,*i)*_ is the radiometrically calibrated reflectance of pixel (x,y) in spectral band i; *DN*_*(x*,*y*,*i)*_ is the digital number of that pixel (x,y) in spectral band i of the mosaic; and *a*_*i*_ and *b*_*i*_ are the slope and intercept of the linear regression model. These calibration equations were used to convert the UAS imagery from DN to reflectance [3].

After radiometric calibration, four different vegetation indices were calculated using the Band Math function in ENVI. These include NDVI, Green NDVI, the enhanced vegetation index (EVI), and the modified triangular vegetation index (MTV12). These vegetation indices are illustrated in the following Eqs (2)–(5):

$$NDVI=\frac{\rho \_NIR-\rho \_Red}{\rho \_NIR+\rho \_Red}$$ (2)

$$Green\;NDVI=\frac{\rho \_NIR-\rho \_Green}{\rho \_NIR+\rho \_Green}$$ (3)

$$EVI=\frac{2.5\times \left(\rho \_\mathrm{NIR}-\rho \_\mathrm{Red}\right)}{\left(1+\rho \_\mathrm{NIR}+6\times \rho \_\mathrm{Red}-7.5\times \rho \_\mathrm{Blue}\right)}$$ (4)

$$MTVI2=\frac{1.5\times \left[1.2\times \left(\rho \_\mathrm{NIR}-\rho \_\mathrm{Green}\right)-2.5\times \left(\rho \_\mathrm{Red}-\rho \_\mathrm{Green}\right)\right]}{\sqrt{\left[{\left(2\times \rho \_\mathrm{NIR}+1\right)}^{2}-\left(6\times \rho_{\mathrm{NIR}}-5\times \sqrt{\rho_{\mathrm{Red}}}\right)-0.5\right]}}$$ (5)

where ρ(Blue), ρ(Green), ρ(Red) and ρ(NIR) are the calibrated reflectance in Blue, Green, Red and NIR spectral bands, respectively. For each field plot, a region of interest (ROI) was established manually by choosing the central two rows and mean value of vegetation indices were extracted corresponding to each plot.

### Field data collection

From each study plot, LAI was measured using a LI-COR LAI-2200C Plant Canopy Analyzer (LI-COR Biosciences., Lincoln, NE, United States) within one day of the UAS flight [22]. To estimate f_c_, overhead photographs were taken using a standard digital camera mounted on a pole positioned approximately 3 m above the ground looking directly down at the plant canopy. Three photos were taken from each plot. Overhead photos were cropped using Adobe Photoshop (Adobe Systems, San Jose, CA, United States) to include central rows from each plot. After cropping, photographs were imported into ENVI for estimating f_c_. The “maximum likehood” supervised classification function was used to classify each image to vegetated and non-vegetated pixels. Then, the “quick stats” function was used to determine the number of pixels in the vegetated areas. Dividing the number of pixels in the vegetated portions by the total number of pixels provided an estimation of f_c_ in each cropped image. Grain yield from each plot was determined after machine harvesting the center two rows. Final grain yields were adjusted to 14% moisture content.

### Statistical analysis

Regression models were developed to predict LAI and f_c_ using NDVI, Green NDVI, EVI and MTVI2 estimated from UAS images. Data from 54 plots over the three UAS image acquisition dates resulted in 162 data points, which was divided randomly into training and testing data sets. We used data from the training data sets for developing regression models. The test data set were used for analyzing the performance of regression models. The performance of regression models in estimating LAI and f_c_ were evaluated by calculating the root mean squared error (RMSE) and the Mean Absolute Performance Error (MAPE). In addition, student's t tests were used to determine if the empirical models (observed vs. predicted) could predict f_c_ and LAI with reasonable accuracy. If the values of slopes are not significantly different from 1 and the values of intercepts are not significantly different from 0, then it can be concluded that the regression was not significantly different from the 1:1 line.

## Results and discussions

### Relationship between vegetation indices and sorghum growth traits

To assess the performance of each vegetation index for estimating LAI and f_**c**_, we first compared the R^2^, RMSE and MAPE of the regression relationships for the training data set (Table 1 and Table 2). The RMSE between NDVI, Green NDVI, EVI and MTVI2 with LAI were 0.28, 0.34, 0.34 and 0.29 respectively. The coefficient of regression (R^2^) of the relationships between NDVI, Green NDVI, EVI and MTVI2 with LAI were 0.91, 0.81, 0.79 and 0.86 respectively. The RMSE between NDVI, Green NDVI, EVI and MTVI2 with f_c_ were 0.059, 0.08, 0.09 and 0.063 respectively with R^2^ of 0.89, 0.78, 0.72 and 0.86. For both LAI and f_c_, NDVI showed the highest R^2^ and the lowest RMSE and MAPE. In our study, EVI showed lower accuracy compared to other vegetation indices although EVI is sensitive to canopy structural variations [24]. This could be because of the coefficients we adopted in the EVI equation [25]. The MTVI2 performed better than Green NDVI and EVI, however this index had low R^2^ and high RMSE and MAPE compared to NDVI. Hence, we chose NDVI for further analysis in this study.

**Table 1:** Regression models developed between vegetation indices and leaf area index (LAI) for the training data set. Best fit functions, determination coefficients (R^2^), root mean square errors (RMSE) and mean absolute performance errors (MAPE) are presented for the four vegetation indices.

Vegetation Index	Regression Model	R^2^	RMSE	MAPE (%)
NDVI	0.14*exp*^(3.4*NDVI)^	0.91	0.28	11
Green NDVI	0.0909*exp*^(3.98*GreenNDVI)^	0.81	0.34	16
EVI	0.567*exp*^(2.21*EVI)^	0.79	0.34	16
MTV12	0.574*exp*^(2.295 *MTV*12)^	0.86	0.29	13

**Table 2:** Regression models developed between vegetation indices and fractional vegetation cover (f_c_) for the training data set. Best fit functions, determination coefficients (R^2^), root mean square errors (RMSE) and mean absolute performance errors (MAPE) are presented for the four vegetation indices.

Vegetation Index	Regression Model	R^2^	RMSE	MAPE (%)
NDVI	1.08 (*NDVI*) − 0.18	0.88	0.06	8
Green NDVI	1.57 (*GreenNDVI*) − 0.54	0.78	0.08	15
EVI	0.59 (*EVI*) − 0.26	0.72	0.06	21
MTV12	0.76 (*MTV*12) − 0.26	0.86	0.06	12

**Fig 2:**
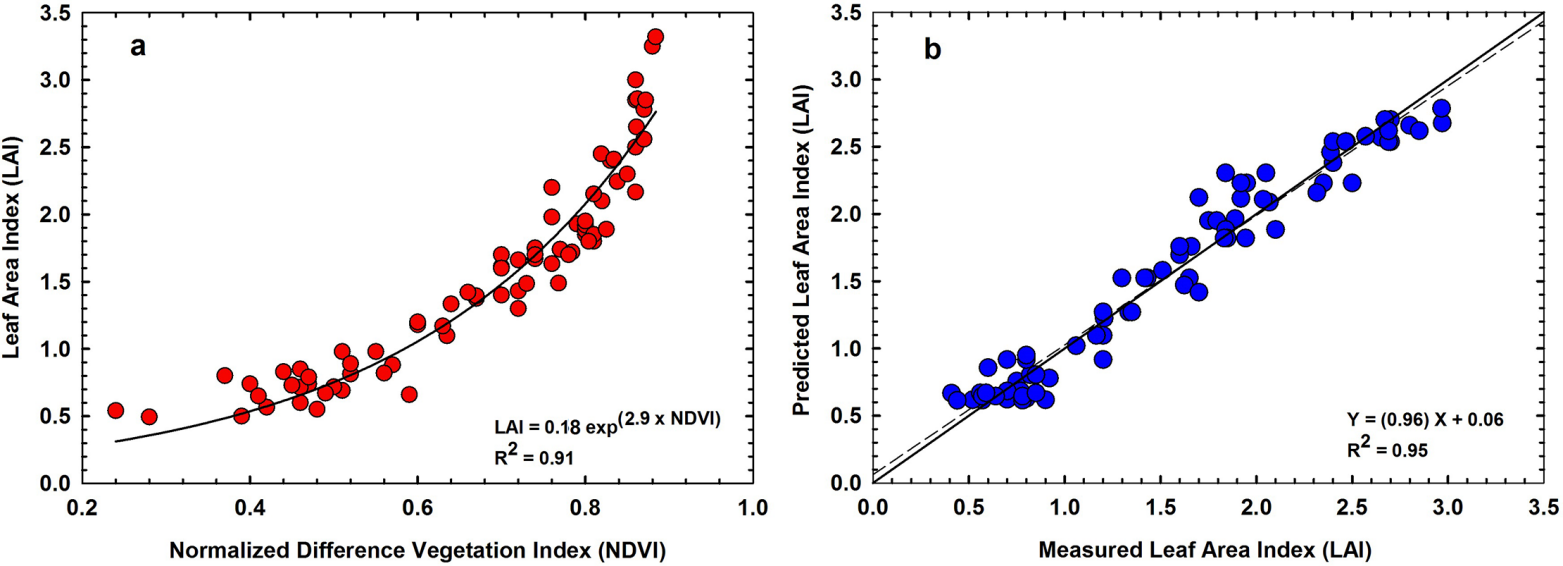
(a) Relationship between normalized difference vegetation index (NDVI) and leaf area index (LAI); (b) Measured LAI vs. corresponding values of LAI predicted using the empirical equation in Fig 2A. The solid black diagonal line in the graph is the 1:1 line. The dashed black line is the least-squares linear regression between the measured and predicted values.

Fig 2A presents NDVI of the training data set plotted against corresponding LAI. The LAI of the hybrids during the measurement period ranged from 0.2 to 3.3. For majority of row crops, as LAI approaches 2.5 or above, canopy reflectance of red light is less than 5% as absorption peaks above 95% [17,18]. Hence, further increases in LAI do not cause significant changes in absorption and reflectance of red light [23, 24,25,26]. This causes NDVI to become invariant to further increases in leaf area development. In order to assess the accuracy and viability of the empirical relationship of NDVI with LAI, we made a cross-validation of measured LAI with the predicted LAI retrieved from the regression model presented in Fig 2A. Fig 2B shows the results of the comparison between measured LAI and predicted LAI retrieved using NDVI from UAS images for the test data set. There was a strong linear relationship between predicted and measured LAI (RMSE of 0.16 and MAPE of 13%) (Fig 2B). The least-squared linear regression equation fit to these points explained 96% of the total variance among the points. Results of a student’s t test showed that the slope was not significantly different from 1 (p = 0.14) and the intercept was not significantly different from 0 (p = 0.15). Thus, we can conclude that the regression line was not significantly different from the 1:1 line. Statistical analysis suggests that the NDVI-LAI regression model did not depend on the choice of the training data set, predicting LAI without a systematic over or under-estimation.

**Fig 3:**
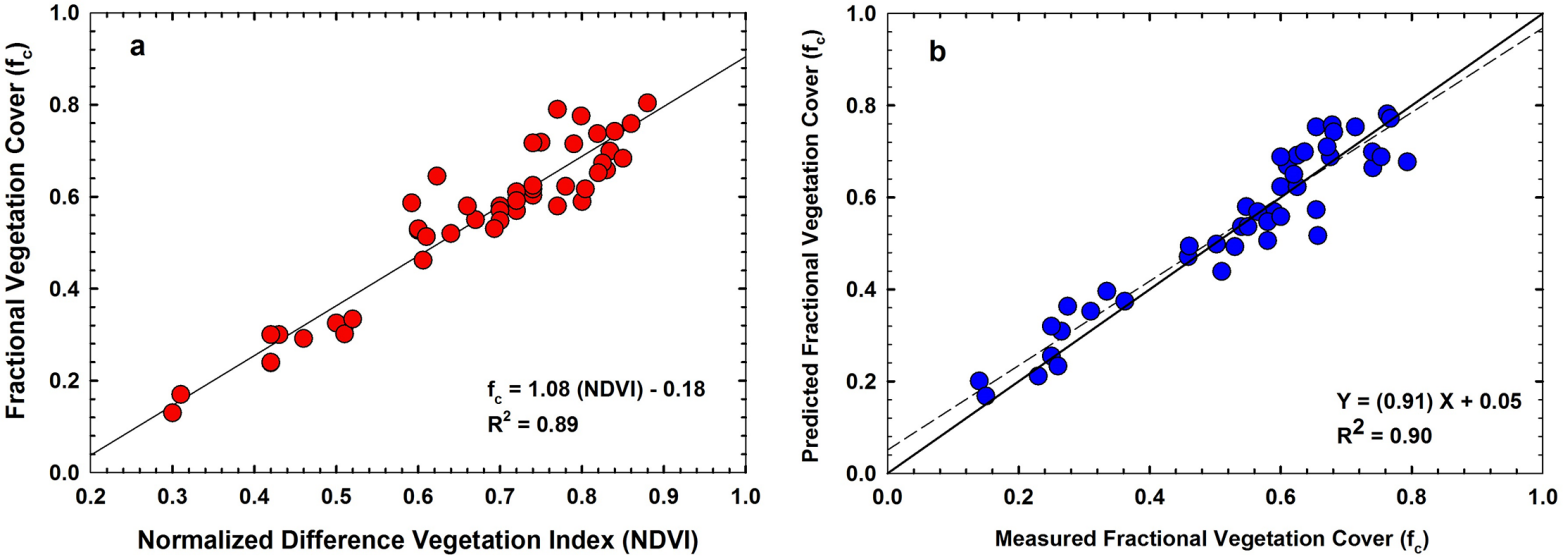
(a) Relationship between normalized difference vegetation index (NDVI) and fraction cover (f_c_); (b) Measured f_c_ vs. corresponding f_c_ values predicted using empirical equation in Fig 3A. The solid black diagonal line in the graph is the 1:1 line. The dashed black line is the least-squares linear regression between the measured and predicted values.

**Fig 4:**
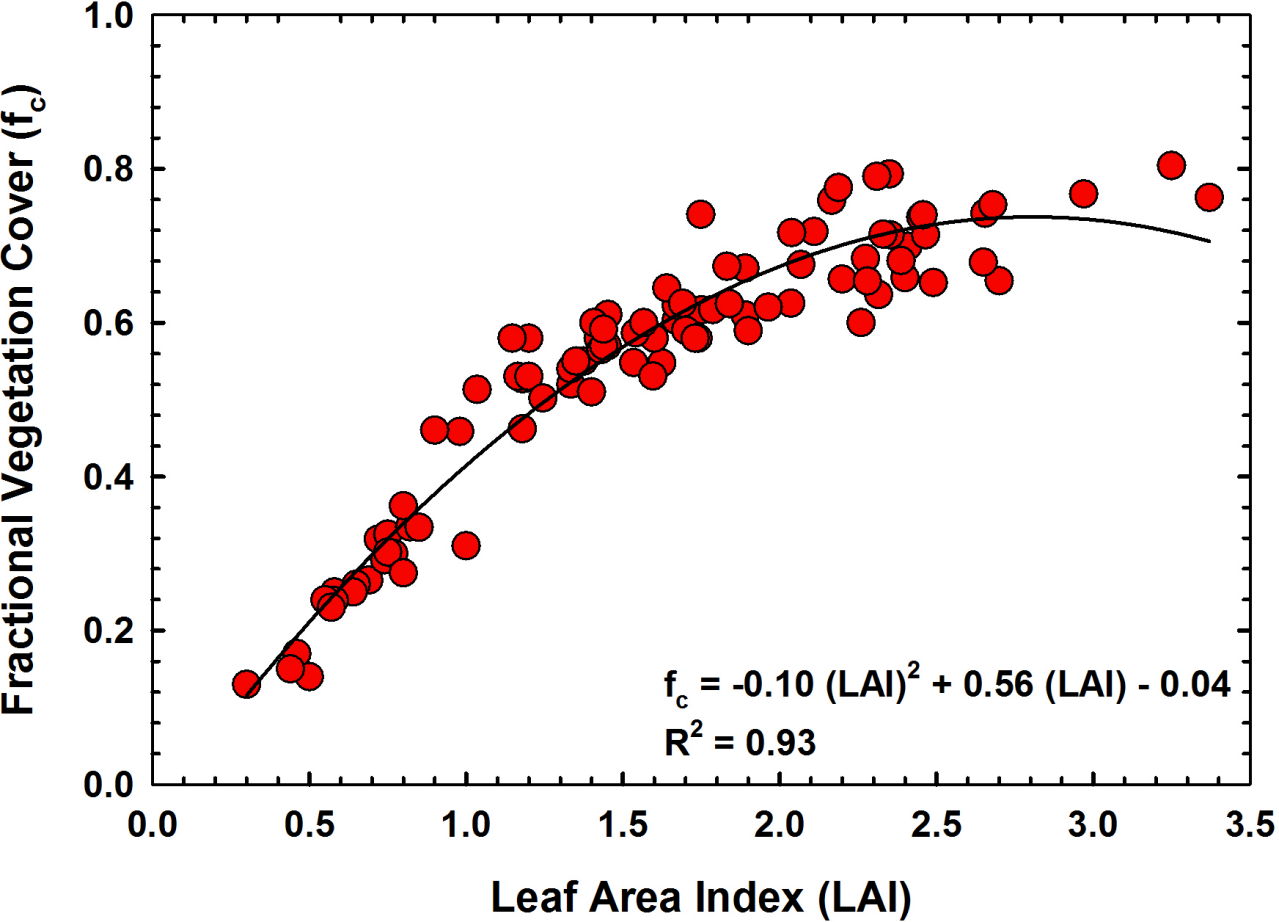
Relationship between leaf area index (LAI) and fraction cover (f_c_) of sorghum.

Fig 3A presents the relationship between NDVI and vegetation cover (R^2^ = 0.89). Unlike the NDVI-LAI relationship, NDVI-f_c_ relationship was linear in nature. This could be expected as the relationship between LAI-f_c_ is curvilinear in nature as demonstrated in Fig 4. Measured LAI values from all study plots were plotted against corresponding f_c_ measurements in Fig 4. As seen in the figure, the sorghum canopy covered approximately 70% of the ground area when it reached an LAI of about 2.5. Further development in leaf area, did not cause any changes in vegetation cover and the relationship became curvilinear in nature. Hence, plots with high vegetation cover will have similar NDVI values although LAI could be still increasing. This will result in a cluster of points at high f_c_ when NDVI is plotted against f_c_ as seen in Fig 3A. Similar to the NDVI-LAI relationship, we made a cross-validation of measured f_c_ with predicted f_c_ retrieved using the regression model presented in Fig 3A. Fig 3B shows the results of the comparison between measured f_c_ and predicted f_c_ using the regression equation in Fig 3A. Fig 3B demonstrates that predicted and measured f_c_ were in good agreement with R^2^ of 0.90, RMSE of 0.05 and MAPE of 4%. The slope of this regression (0.91) was close to 1, while the intercept (0.05) was close to zero. Results of a student’s t test showed that this slope was not significantly different from 1 (p = 0.07 and the intercept was not significantly different from 0 (p = 0.05).

**Fig 5:**
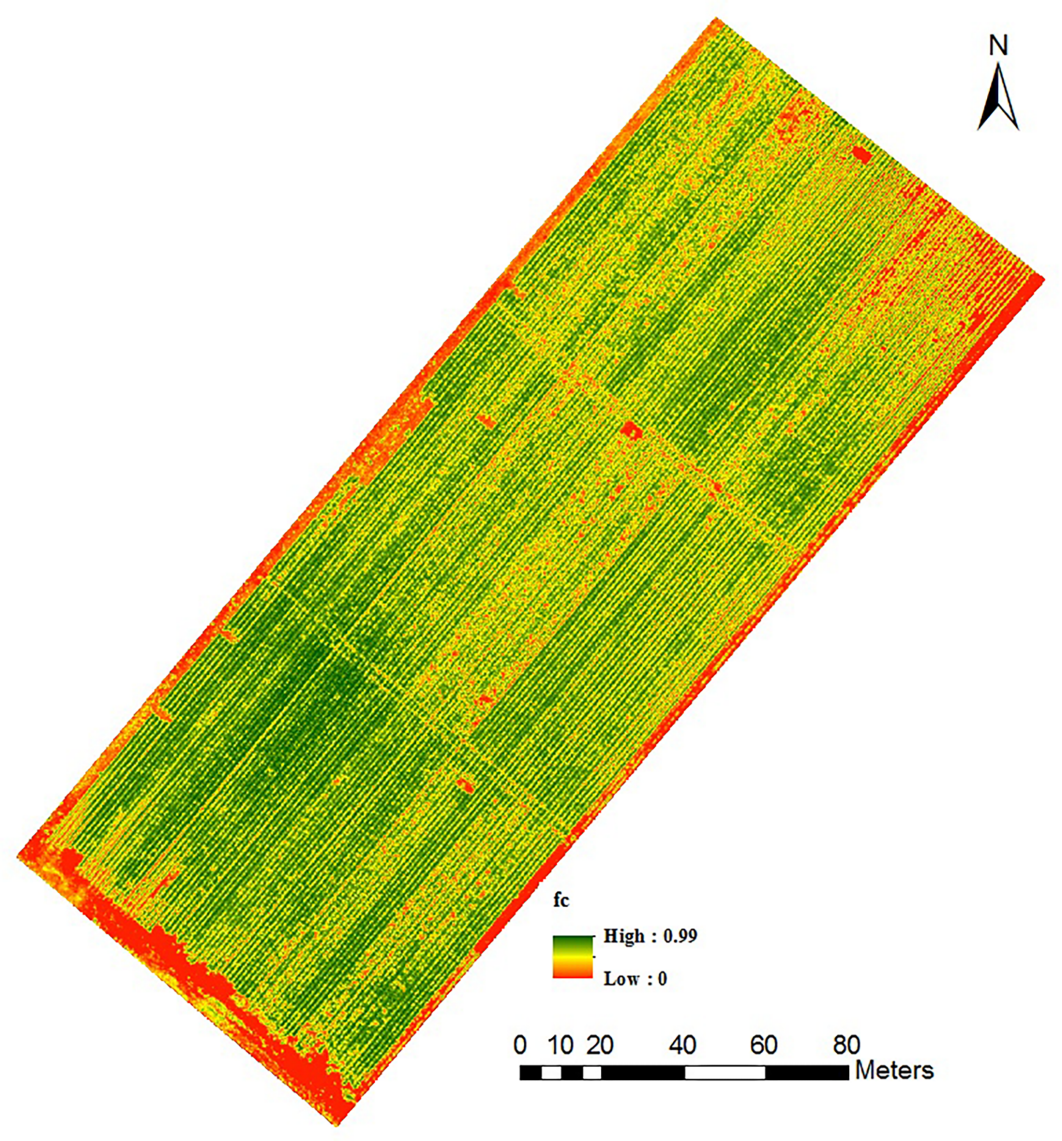
Fractional vegetation cover (f_c_) map of the sorghum field derived from UAS imagery acquired on 10 June 2016.

The results from our study showed that empirical relationships of vegetation indices and crop biophysical parameters are useful for predicting within-season crop growth traits. The type of NDVI-f_c_ and NDVI-LAI relationships that we observed in our study is consistent with previous studies that reported linear relationships between f_c_ and NDVI and exponential relationships between LAI and NDVI [27–31]. The NDVI saturation issue can reduce its functionality for LAI prediction at very high LAI values. However, NDVI is still one of the most widely used vegetation indices to predict LAI and f_c_ from remotely sensed data especially from early to mid-growing season. Reliable empirical relations developed using within-season data such as in this study can be used to convert UAS multispectral imagery into maps of plant physiological properties. For example, Fig 5 shows the f_c_ map for the study field which was developed using UAS image data acquired on 10 June 2016. The f_c_ map of this sorghum field showed considerable variations in vegetation cover. The areas with dark green color show dense canopy while the areas with red to yellow color shows sparse canopy, which represents bare soil or plots with low seeding rates. These types of maps have particular applications in plant breeding research. The number of plots in a plant breeding program at a single location can range from few hundred to several thousands in a single growing season [32]. With-in season empirical relationships developed and validated using a smaller number of plots could be applied to map and quantify plant physiological properties for the remaining larger number of plots. However, caution should be taken before applying the empirical relations for extrapolation to areas other than where it was developed. Changes in soil characteristics may require the development of newer algorithms. Similarly, temporal stability of these relationships over multiple growing seasons require further investigations.

### NDVI and seeding rates

**Fig 6:**
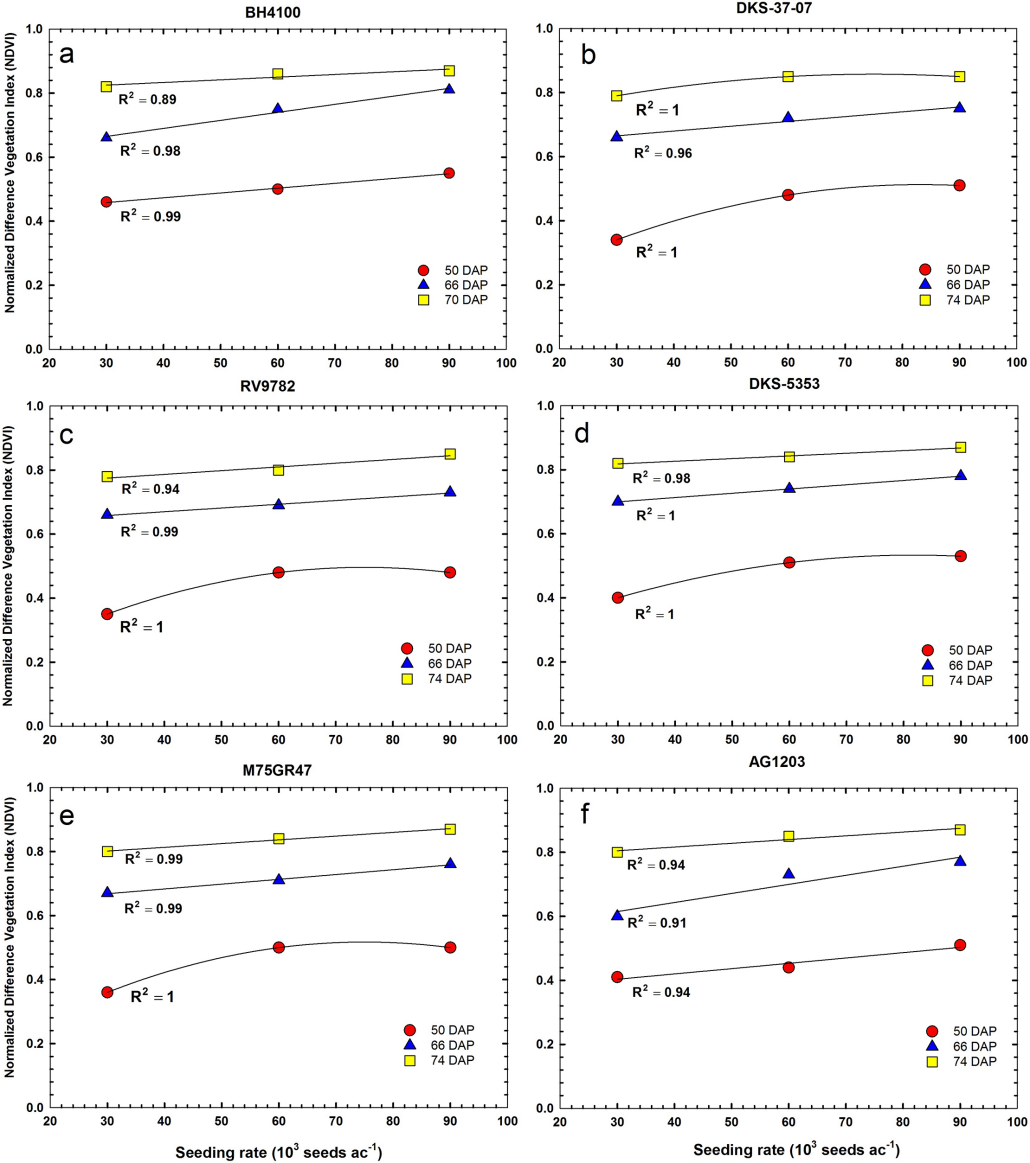
Relationships between normalized difference vegetation index (NDVI) and seeding rates for six different sorghum hybrids at 50, 66 and 74 days after planting (DAP) in 2016. Each data point represents the mean of three replicates and was regressed against seeding rate.

Fig 6 shows the responses of NDVI to seeding rates for six sorghum hybrids at three different dates of UAS image acquisition. In general, there was a significant positive correlation between NDVI and seeding rates for all sorghum hybrids. The relationships between NDVI and seeding rates were linear or quadratic in nature (Fig 6). Positive linear relationships were more frequently observed between NDVI and seeding rate when plant NDVI was relatively higher. Early in the season when plant NDVI was low (50 DAP), quadratic relationships were more pronounced. The average NDVI value at 50 DAP was 0.39 at the low seeding rate (30,000 ha^-1^). NDVI increased to an average of 0.49 and 0.51 for the medium (60,000 ha^-1^) and high (90,000 ha^-1^) seeding rates, respectively. The average NDVI increased to 0.66, 0.72 and 0.77 at 66 DAP for the low, medium and high seeding rates, respectively. The average NDVI at 74 DAP was 0.80, 0.84 and 0.86, for the low, medium and high seeding rates, respectively. As the results in our study show, a lower seeding rate may not lead to low NDVI compared to medium and high seeding rates. When sorghum is planted at a lower density, the additional spacing between plants may trigger formation of new, well-developed tillers as the season progress [33]. In our study, difference in NDVI between seeding rates were more pronounced at 50 DAP and 66 DAP. Hence early season NDVI measurements could be a useful index for estimating plant population density. This agrees with previous research results involving field-based remote sensing measurements [34,35].

### Sorghum yield

**Fig 7:**
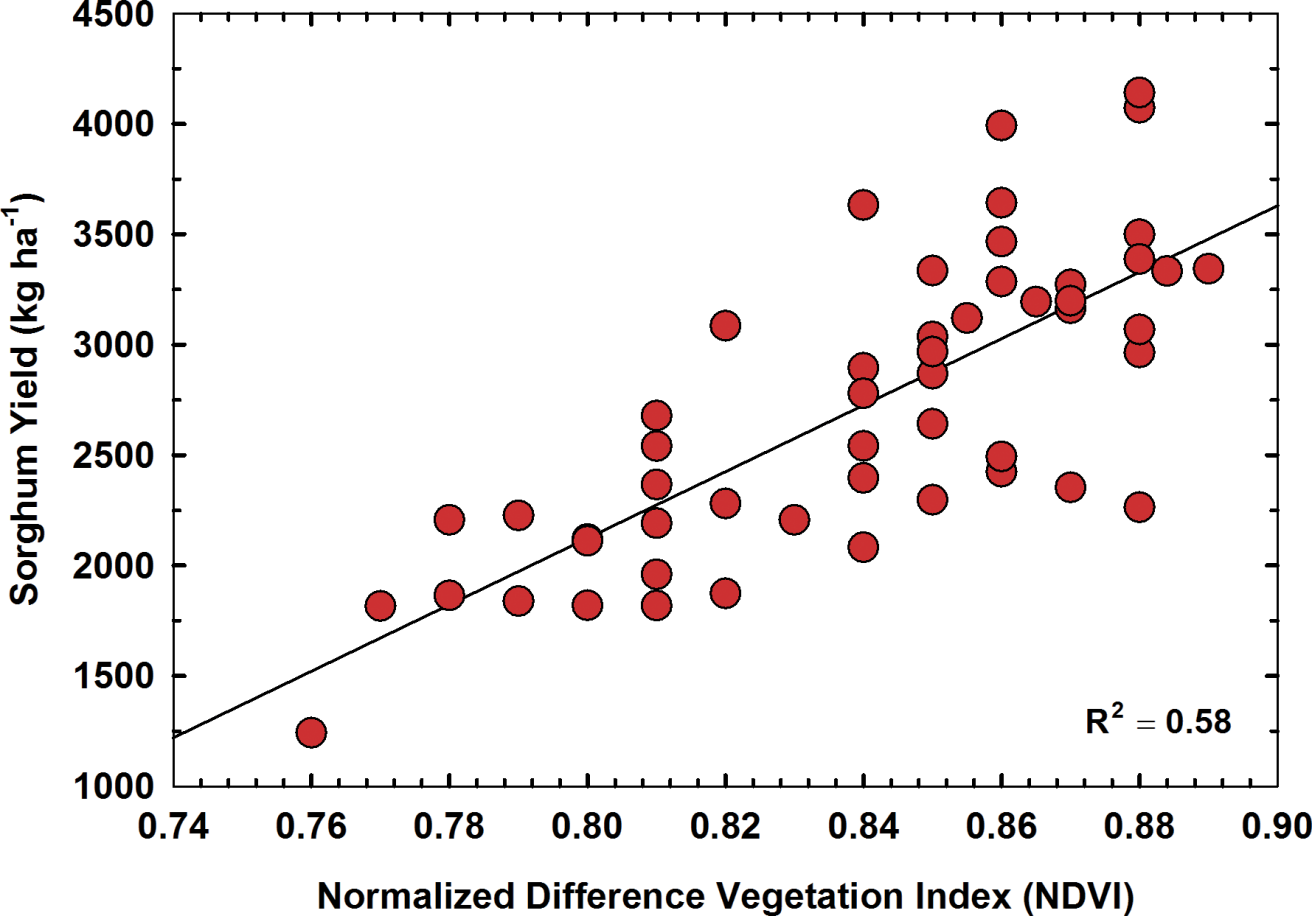
Relationship between final sorghum yield and NDVI on 18 June 2016.

The average sorghum yield in our study was 2,720 kg ha^-1^, with the lowest yield of 1,242 kg ha^-1^ and the highest yield of 4,144 kg ha^-1^. The final sorghum yield was correlated with three different NDVI data sets extracted from UAS images acquired on 24 May, 10 June and 18 June, 2016. The statistical results showed that NDVI values calculated for 18 June had the highest correlation with final sorghum yield indicating imagery taken at this particular growth stage (flowering) could be a better indicator of yield (Fig 7). However, the R^2^ value for the linear regression between NDVI and grain yield was 0.58 for this analysis due to variabilities in harvested grain yield caused by inclement weather conditions at the time of harvest. Frequent precipitation (approximately 228 mm) received towards the end of the growing season delayed harvesting of the study plots. Frequent precipitation and humid weather conditions immediately prior to harvest had caused grain mold disease and impacted yield to some extent in our study. Similar issues with grain mold disease and yield reduction in sorghum had been reported by other researchers [36,37]. Measurements of leaf chlorophyll content, plant height, and plant density using UAS data could be incorporated in yield prediction models to improve the accuracy of yield estimation. However, such an analysis was outside the scope of this study.

### Conclusions

Results presented in this paper show that high resolution images acquired using UAS can be used effectively for within-season data collection from agricultural fields. We used a cost-effective multi-spectral sensor mounted on a fixed-wing UAS for collecting image data of six sorghum hybrids planted at three different seeding rates during the 2016 growing season. Three UAS flights were carried out in the growing season. The relationship between NDVI and LAI and between NDVI and f_c_ were validated and proved to be robust for estimating LAI and f_c_ from UAS derived NDVI values. NDVI obtained for 18 June 2016 (74 DAP) was found to be best correlated with final grain yield, indicating imagery taken at flowering stage could be a better indicator of yield rather than NDVI obtain at earlier growth stage of sorghum crop. Our results also showed that early season NDVI measurements could be a useful index for estimating plant population density in sorghum. The observed high correlation between UAS-derived NDVI with f_c_, LAI and grain yield indicates the applicability of UAS for within-season data collection of agricultural fields. Future work will focus on additional experiments using different sensors to investigate the possibility of estimation of different agronomic parameters through various other indices for greater precision in crop monitoring.

## Supporting information

S1 Table Data used for developing regression models between vegetation indices and leaf area index (LAI) for the training data set. (XLSX)

S1 Fig Normalized difference vegetation index (NDVI) and leaf area index (LAI) data. (XLSX)

S2 Fig Normalized difference vegetation index (NDVI) and fraction cover (fc). (XLSX)

S3 Fig Leaf area index (LAI) and fraction cover (fc). (XLSX)

S4 Fig Raw UAV data. (ZIP)

S5 Fig Normalized difference vegetation index (NDVI) and seeding rates. (XLSX)

S6 Fig Normalized difference vegetation index (NDVI) and yield. (XLSX)
